# Sex determination strategies in 2012: towards a common regulatory model?

**DOI:** 10.1186/1477-7827-10-13

**Published:** 2012-02-22

**Authors:** Roxani Angelopoulou, Giagkos Lavranos, Panagiota Manolakou

**Affiliations:** 1 Experimental Embryology Unit, Department of Histology and Embryology, Medical School, Athens University, Athens, Greece

## Abstract

Sex determination is a complicated process involving large-scale modifications in gene expression affecting virtually every tissue in the body. Although the evolutionary origin of sex remains controversial, there is little doubt that it has developed as a process of optimizing metabolic control, as well as developmental and reproductive functions within a given setting of limited resources and environmental pressure. Evidence from various model organisms supports the view that sex determination may occur as a result of direct environmental induction or genetic regulation. The first process has been well documented in reptiles and fish, while the second is the classic case for avian species and mammals. Both of the latter have developed a variety of sex-specific/sex-related genes, which ultimately form a complete chromosome pair (sex chromosomes/gonosomes). Interestingly, combinations of environmental and genetic mechanisms have been described among different classes of animals, thus rendering the possibility of a unidirectional continuous evolutionary process from the one type of mechanism to the other unlikely. On the other hand, common elements appear throughout the animal kingdom, with regard to a) conserved key genes and b) a central role of sex steroid control as a prerequisite for ultimately normal sex differentiation. Studies in invertebrates also indicate a role of epigenetic chromatin modification, particularly with regard to alternative splicing options. This review summarizes current evidence from research in this hot field and signifies the need for further study of both normal hormonal regulators of sexual phenotype and patterns of environmental disruption.

## Background

Sex is believed to be a complex regulatory model which involves the fine-tuned action of numerous genes affecting most aspects of an organism's functional systems. Rather than simply providing a solution in a species' need for reproductive survival, sex has been shown to be a much more intriguing phenomenon, directly controlling major morphological and physiological processes, such as development, differentiation and metabolism. This reality has also lead to the adaptation of the term "sexual dimorphism" in species with a male-female sex pattern, in order to describe the complete set of structural and functional changes involved in the establishment of the sexual phenotype. Indeed, the ability to develop and maintain what science currently perceives as "normal" sex basically implies a co-operation of various genes whose expression is induced or inhibited at preset crucial time periods by a combination of genetic and epigenetic control elements [1]. These elements may themselves be the direct target of hormonal (e.g. sex steroid) action or, alternatively, they might be affected by various mediators within the cellular microenvironment, which, depending on the species, reflect different environmental adaptations (e.g. temperature, nutrients) [2]. The temporal regulation of sexual phenotype is by itself a hot research field, since sex-related changes have been shown to pursuit not only during fetal development or early childhood, but virtually throughout life, including adult years and, eventually, reproductive senescence [3].

Since the evolution of such a complex regulatory system would require significant energy resources (to cover the production of the various mediators and modulators of gene action and their distribution in the different tissues and organs) one might assume a need for a justification for its evolutionary maintenance within the global context of cellular economy. Indeed, it is likely that sex has developed in the course of evolutionary history as a mechanism of improved energy distribution and increase in phenotypic variance, a process that might be traced back to the early onset of cellular aerobic metabolism and reactive oxygen species production (via selective pressure) [4]. In particular, qualities which might provide a potential survival advantage could accumulate via the process of natural selection, in a context of constant dynamic environmental change and limitation of available resources. Moreover, the ultimate phenotype of a species may also be affected by sex selection, a continuing process which also causes continuous evolutionary pressure [5]. In this aspect, a sexual phenotype which allows to distribute resources among terminally differentiated tissues efficiently, to coordinate their action according to the given conditions and to increase variance via the production of derivative cells with different combinations of characteristics compared to the original organism seems to be an effective survival strategy [3,6].

The next step in the attempt to understand sex in general could be the search for those conditions that may grossly affect survival and are therefore candidates for regulation both upstream (i.e. primary sex determination) and downstream (i.e. as targets for the differentiation of sexually dimorphic traits). Although one might argue that this pursuit is more philosophical than purely scientific and that the answer might not be the same for all life species, there is little doubt that some basic factors have remained crucial throughout the evolutionary process. These definitely include extreme weather conditions (temperature/heat, access to water), nutritional limitations and protection from predators/antagonistic species. All these circumstances reflect an unstable environment which places different priorities at various times [1,2,7]. Thus, it seems reasonable to suppose that sex itself must have evolved within this context, expanding its actions to include beneficial physiological adaptations according to the demands of every given space and time period. Within this perspective, sexual phenotypes in their current form are a kind of viable "fossil" by themselves, incorporating and reflecting evolutionary history and major selection events in each species' background.

**Figure 1:**
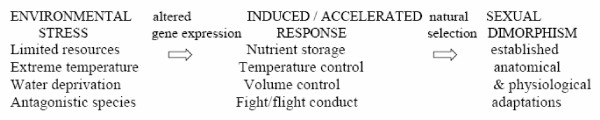
**Sex evolution as a response to environmental stress**. Although the exact mechanisms may differ among species, the rationale remains the same, placing environment as the generator of natural selection and sex as a strategy to improve survival potential. Note that the term "induced response" doesn't imply direct functional change in response to the changing environment (as Jean Baptiste Lamarck suggested) but rather a promotion of phenotypes produced via random events (e.g. mutations).

The aforementioned short description of the proposed origin and development of sex as a survival tool results in two subsequent conclusions. First, it implies that different species would be expected to present with different spectrums of sex specific functions, since the conditions and challenges in the past that have lead to their modern form were also distinct (thus resulting in the need for specific regulatory adaptations in terms of anatomy and physiology). This is indeed true and a study of the sexual phenotype in nature clearly identifies different levels of dimorphism with regard to diverse qualities, such as skin color and texture, voice, hair, muscle and fat distribution, reproductive organs, motility and brain function, to name but a few examples [2,3]. The other conclusion is that despite the details pertaining to each organism, a variable combination of environmental, genetic-epigenetic and hormonal elements must have guided any specie's sexual phenotype evolution to this date and still provide the driving force for future adaptations in its sexual dimorphism (Figure 1) [7-9].

The authors have already contributed in this field by an extensive review paper published in *RBEJ *in 2006, which described the underlying molecular mechanisms of sex determination in a variety of model organisms. Six years later and in response to the warm reception of the original manuscript by the audience (highly cited and highly accessed on a weekly basis ever since) the authors have been invited to attempt a short update based on research data accumulated in the meantime, which has resulted in the production of the current contribution.

## Sex determination pathways: Basic terminology

Although both inherent and environmental elements contribute to sex selection and determination in most species, the basic regulatory mechanisms are traditionally described as environmental sex determination (ESD) and genetic sex determination (GSD) [10]. The former refers to species in which a direct association between specific environmental conditions (temperature, nutrients) and offspring sex has been observed [10,11]. This also implies that the sex of the embryo cannot be determined in advance (upon conception), but rather arises subsequently as the best choice given specific external conditions during a critical sex determination stage, early in its development [11,12]. In the latter case, i.e. GSD, this is not true and the embryo's sex is regulated by genes transferred by its parents during conception. This observation indicates a preexistent commitment to a specific sex, which cannot normally be reversed later on (although extreme environmental disruptors or hormonal manipulations may actually cause some degree of sexual phenotype reversal) [10,13].

Another important distinction is that between primary and secondary (subsequent) sex determination. In effect, primary sex determination refers to the chronologically first evident expression of sex--specific functions and structures in a given species. For instance in mammals, this corresponds to *SRY *expression in chromosome Y, leading to the differentiation of the gonadal primordium to the formation of a testis (or, alternatively, lack of its expression and differentiation to an ovary) [10,13]. On the other hand, secondary (subsequent) sex determination (or sex differentiation) refers to sex--specific functions and structures affecting a number of tissues and organs, if not the whole body. It is significant to note that in invertebrates (worms, insects), primary sex determination seems to refer simultaneously to cells along the whole body, rather than a specific organ, making the distinction between primary and secondary determination less applicable [14]. It is also interesting that recent data on avian sex determination seem to indicate that a single-cell sex selection process across the body may exist in such species as well (see relevant section) [15]. Henceforth, the use of the term sex determination will be used to refer exclusively to primary selection, while secondary determination will be referred as sex differentiation.

Another significant player in sex determination are the so-called sex steroids. This term refers to any sterol derivatives which are either expressed differentially in males and females or, have the ability to cause sex--specific reactions when administered externally [16]. This broad definition doesn't demand a specific role for sex steroids in the normal regulatory system of sex determination. Indeed, while the latter has been clearly shown and proven for certain species (e.g. mammals, reptiles) [10-13,16], it is less clear for other organisms (e.g. insects) [14]. This may reflect differences in terms of specific steroid molecules, steroid receptors and other nuclear receptor families in various species.

If sex has indeed evolved as an evolutionary mechanism to improve energy storage and distribution via selective steroid expression and function, one would expect sex--specific steroid action as a core element in all sex determination systems [16]. However, such a global effect has not been proven so far and therefore remains an open question. Moreover, sex steroid action may be placed at a different position in the chain of events leading to the establishment of the sexual phenotype in various species [10]. For instance, in many reptiles, regulation of aromatase expression is the central phenomenon leading to sex determination [11,12] while in mammals, sex steroids are only involved in subsequent sex differentiation [10,16]. For the purposes of this review, sex steroid action is basically discussed with regard to sex determination only (whenever applicable)[Table 1].

**Table 1:** Regulatory elements in sex determination/dosage compensation

Species	Worms	Insects	Reptiles	Amphibians	Fish	Birds	Mammals
**Epigenetic phenomena**	unknown	alternative splicing	unknown	unknown	unknown	unknown	X inactivation
**Primary sex determination**	cellular	all tissues	gonad	gonad	gonad	all tissues	gonad
**Role of sex steroids**	unclear	unclear	sex det.	sex diff.	sex diff.	sex diff.	sex diff.
**Role of Temperature**	unclear	TSD (rare)	TSD	TSD	TSD	none	none
**Sex chromosomes**	X, W	X(Y) ZZ/ZW	ZZ/ZW XX/XY	ZZ/ZW (*Xenopus*)	XX/XY ZZ/ZW	ZZ/ZW	XX/XY
**Sex determining gene(s)**	her-1, fem, tra	SXL	no	DM-W	DMY	DMRT1	SRY

Major regulatory elements in sex determination systems across the animal kingdom. Extensive similarities are noted even among distant species. Sex det: sex determination, Sex diff: Sex differentiation TSD: temperature-sensitive sex determination

## Environmental sex determination (ESD)

The previous section has already introduced the role of the environment in sex evolution. However, an interesting discussion arises when one attempts to examine its role in sex determination and differentiation as they are observed today. In this case, research would seem to indicate that while environmental control is clearly demonstrated in certain species (reptiles, amphibians and fish) with regard to either temperature conditions and/or nutrients in the microenvironment, this is not equally evident in others (birds, mammals) [1,2]. In the latter case, sex seems to be a more predetermined, genetic (chromosomal) event rather than an externally inducible one, although even in these organisms, extreme exposure to sex steroids may largely differentiate an established sexual phenotype (without allowing fertility, should this occur in a post-development stage) [6]. Therefore, it might be reasonable to assume that hormones, especially sex steroids, retain their role as key mediators of sex differentiation in response to environmental conditions, possibly providing the missing link between them [16]. This association could become stronger when more model organisms are examined and the role of steroids in the sexual maturation of various species is definitely proven, as is already the case for a significant number of mammals, reptiles and birds [10,16].

The best known example of ESD to date is temperature-dependent sex determination or TSD [11,12]. In this case, sex determination is based on environmental temperature in the area immediately surrounding the egg/embryo during a crucial developmental stage. The temperature range within which sex shifts towards the male or female differs considerably among species, as well as the "hierarchical" placement of the 2 sexes (i.e. whether the male or female is preferred in higher temperatures) [11,12,17]. With regard to target molecule, most data indicate a direct effect of temperature on the expression of the aromatase enzyme complex, which is associated with estrogen production and action [11]. However, this has not so far been proven in all TSD species and therefore alternative end-points may also exist [10,11].

## Genotypic sex determination (GSD)

The majority of vertebrates are known to possess genes that are exclusively or at least differentially expressed in one sex in proportion to the other [13]. In some cases, a whole pair of chromosomes is attributed to sex-related content, in which case they are named sex chromosomes or gonosomes (contrary to the remaining homologous pairs, or autosomes). Interestingly, even in the case of distinct sex chromosomes (XX/XY or ZZ/ZW respectively), some areas retain homology and usually mediate non-sex related functions (pseudoautosomal regions, involved in chromosomal pairing during meiosis) [10]. On the other hand, several genes located on the sex chromosomes have not been shown to be directly related to sex determination and differentiation. Thus sex chromosomes actually contain both sex-related and non sex-related genes [10,13]. Finally, these processes involve a massive reorganization of chromatin status (X chromosome inactivation, dosage compensation) and multiple downstream gene actions, which also include the selective expression of genes located on autosomes [10]. Therefore, GSD is by no means a case of a single-gene controlled trait but rather a delicate interaction of multiple genes, one or several of which might code for the initial trigger that generates the cascade of events leading to permanent sexual phenotype establishment. The latter also requires the active participation of sex steroids and their receptors, as well as various other mediators at a tissue- and time- specific level [1,7,16].

The previous units have already included selected examples of organisms which feature elements of GSD or even complete sex chromosomes. Indeed, this is true for a wide range of animals, including representatives of all major classes of both invertebrates and vertebrates [3,8,10,13,14,18,19]. An important subsequent division, however, lies on the deterministic nature of sex chromosome presence and sex-related gene expression. For instance, the significance of environmental factors in sex determination in several kinds of fish and reptiles (e.g. turtles) is so great that it practically imposes its selection on any genetic predisposition, shifting the phenotype towards the sex that is preferable in the given conditions [8,12,16-18]. On the other hand, key gene expression in birds and mammals (which are typical examples of GSD) is indeed a deterministic event to select their sexual identity, since it cannot normally be subsequently overrun by external factors and pursue the alternative sex phenotype [2,10,13]. In rare cases in which downstream genetic pathways have been distorted (e.g. gene mutations, hormonal imbalance, receptor resistance), the individual may result with mixed gender characteristics and an almost definitely compromised fertility [1].

## Reptiles: "pure" ESD... and yet not so pure

Perhaps reptiles are the best known and most studied example of ESD [2,11]. In particular, reptiles have been known to exhibit temperature-dependent regulation of sex selection via selective sex steroid production and action for several decades [11,12]. Based on research outcomes accumulated in the last decade, ESD is believed to exist in its most "pure" (i.e. absolute, non-genetic) form in tuatara, where no sex chromosomes have been observed and no major genetic regulatory mechanisms (in the sense of key upstream determinants) have been proposed [18]. A similar pattern has been observed for a number of turtle species and crocodiles [2,11]. On the contrary, other kinds of reptiles exhibit a more complicated regulatory model, with the presence of distinct sex chromosomes (snakes, lizards), either in a XX/XY or a ZZ/ZW pattern, that incorporate several conserved sex-related genes, such as *DMRT1 or SOX9*) [20,21]. However, there is evidence that even in some such cases (e.g. lizards), sex determination is strongly related to environmental status and the final phenotype is more proportional to temperature conditions than actual genetic/chromosome content [17]. Interestingly, the mechanisms of sex determination are extremely diverse even among closely related reptile species, as is the case with geckos [22]. The latter have been shown to present all variants of sex determination patterns, ranging from "classic" ESD-TSD to an intermediate system where temperature is involved in gene expression modification and even to whole sex chromosome regulation [22]. Since steroid expression modification (androgen + estrogen) is a common end-stage control pattern in all these cases, it appears that different pathways may have evolved according to local environmental stress factors and conditions, but the larger picture (i.e. optimal adaptation to existing conditions via hormonal modification) remains basically unchanged [18].

## Amphibians: More evidence needed

Although available data is more limited, the current understanding of sex determination in amphibians is relevantly close to that observed in reptiles [10,18]. Sex chromosomes are a more stable finding in this group, but their type (XX/XY or ZZ/ZW) and gene content differ considerably [10,13]. No definite major sex-determining gene has been described so far, despite retained expression of some conserved sex-related genes, such as *sox3*, which is exclusively expressed in the ovaries of *Xenopus laevis *[23] and *DM-W*, a *DMRT1 *homologue gene on the W chromosome of the same species [24,25]. In the latter case, the significant homology between the genes found in chicken (*DMRT1*) [26,27], frogs (*DM-W*) [13] and the teleost fish medaka (*DMY*) [28] seems to indicate a common origin via duplication and transfer to alternative positions in the genome [13]. Although further details concerning its function in sex determination in *Xenopus *are largely absent, it seems that *DM-W *is a strong candidate for a key regulator of sex determination, as is already known to be the case for its paralogues in the other species mentioned [13]. Thus, the current hypothesis states that the presence of DM-W (and, therefore, a W chromosome) triggers a pathway leading to the development of a female phenotype, while its absence (implying a ZZ genotype) is consistent with a male sex determination [29]. *SOX-3*, a gene bearing homology to the mammalian *SRY *is expressed downstream in the developing ovary and may therefore be one of the subsequent players involved in the establishment of the female phenotype [13,23].

With regard to TSD, the role of temperature in the establishment of the final sex phenotype and its ability to cause sex reversal and surpass genetic predisposition has been shown in some amphibian species, although this may not necessarily be a universal phenomenon [3,19,30]. In particular, classic experiments involving exposure to estrogens during a crucial developmental period have proven the ability to reverse the sexual phenotype, but this process has been proposed to act at the level of subsequent sex differentiation rather than primary sex determination, since in this case the latter appears to remain a genetically predetermined event [19,30].

## Sex determination in fish: A little bit of everything?

Fish are the most variable animal group in terms of sex determination strategies, with experimental data proving largely differential mechanisms among various species. The latter include a combination of environmental and genetic regulatory elements, while many details still remain unclear [8,9].

Environmental control of sex determination in fish may refer to temperature changes, water conditions, nutrients and _P_H, with the biggest quantity of currently available data referring to cases of temperature-dependent sex determination (TSD) [2,8,9]. Interestingly, the temperature range within which a male or female fish evolves differs considerably among various species, so that the highest temperatures might be consistent with both male (most common) and female offspring, depending on species' habitat and life cycle particularities [2,7-9]. Evidently, the achievement and preservation of fertility in fish implies as a prerequisite that temperature control is applied specifically during a specific crucial developmental stage [7-9]. In some cases, experimental data have also demonstrated distortion of the adult sexual phenotype by exposure to extreme temperature conditions, although it is unclear whether this significant in actual life conditions [8,9]. Although alternative explanations may also exist, temperature-related control of P450 aromatase expression and action remains the best studied model via which TSD is proposed to operate in these cases (a concept deriving from similar previous observations in reptiles) [2].

However and despite the definite role of environment and especially temperature in sex selection, many kinds of fish have developed a full range genetic control system for sex determination, including the presence of complete sex chromosomes (XX-XY or ZZ-ZW pairs) [7-9]. Unfortunately, it has not been possible to demonstrate a common pattern of sex determination so far, with an inability to present a specific central genetic regulatory component in most of the fish series studied [7-9]. A significant exception in this field is the recent progress in the study of sex determination in the teleost medaka [28,31,32]. In this model organism, sex is determined via the presence of an XY chromosome pair for males or an XX set for females [31]. The sex-determining gene on the Y chromosome has now been recognized as *DMY*, a homologue to *DMRT1*, a gene with known sex-specific expression in various other species, including mammals and avians [28,31,32]. Interestingly, *DMRT1 *itself is also expressed in the medaka and its normal function has been shown to be necessary to establish the male phenotype, once *DMY *has completed its role as the primary sex determining factor [31,32].

Latest research has also provided evidence that the spectrum of functions involved in sexual dimorphism in fish is not directly proportional to the organism's size, taxonomy or current living conditions [8,33,34]. For example, research in the rainbow trout, *Oncorhynhus mykiss *revealed numerous sex-specific gene expression differences throughout the genome [35,36]. These differences were proportionally different in various tissue samples examined, but remained present in virtually every organ of the body (such as the liver and brain) [36]. Although the role and function of every gene in this series is currently not known, the results clearly indicate the expansion of sexual dimorphism far beyond the conventional range of sex determining genes and reproductive organ formation [36]. With regard to known specific sex-determination genes, *ovol1 *appears to retain a different pattern of expression among males and females, as is also the case in fruitflies and sheep, while others (e.g. *sox9, wt1, DMRT1*) don't seem to have such a role in this species [35-37]. In conclusion, for the majority of fish species, so far it remains unclear whether there is an upstream key sex determination gene or simply a network of interactions, which involves several genes and environmental parameters as well [2,8].

## Avian sex: From gonad to whole--body level

Birds have been shown to present fully-formed sex chromosomes, in a manner similar to mammals, regardless of the fact that there is on-going controversy as to whether there is actual evolutionary connection between the sex chromosomes in these 2 animal groups or not [13,26,27]. Therefore, there is little doubt that genetic control elements are strong determinants of sexual fate in birds [26,38]. Although few model organisms have so far been examined (most currently available data deriving from chicken and zebra finch), it appears that a major regulatory gene may be present at the top of the gene cascade leading to sexual determination and differentiation [10,26,38]. For sexual dimorphism at the gonadal level in chicken, this role seems to be attributed to the expression of *DMRT1*[26]. Interestingly, recent data seems to suggest that this is in fact not a whole-body event but rather a tissue-specific reaction [15,38]. Therefore, if this is indeed the primary sex determination, any other downstream genes must be responsible for the subsequent establishment of the sex phenotype in the various tissues at the body (i.e. sex differentiation) [15,38]. In this context, sexual phenotype may be regionally diverted due to specific local "environmental" interactions affecting *DMRT1 *expression or its downstream cascade [10,15,38]. The latter process may refer particularly to selective exposure to sex steroids or other mediators and is consistent with known primitive strategies of sex selection at an individual cell level, as is the case, for instance, in nematodes such as *C. elegans *[38].

Interestingly, recent research data in monotremes seems to indicate that there is a strong homology between their sex chromosomes (which are mostly a homologous pair, with the exception of the *SRY *region) and the ZZ/ZW pairs of certain avian species (along with certain elements deriving from autosomes) [13,27,38,39]. Although the available evidence is not conclusive, this seems to indicate a potential continuity in evolutionary history, in the sense that the rise of a novel key regulatory gene (namely *SRY*) has been combined with numerous structural and functional adaptations that were pre-existing in a possible common ancestor of birds and mammals to create transient sex determination models, later substituted by the more complex networks involved in modern avian and mammalian sex determination [39]. The latter is characterized by a conserved main gene cassette in both eutherian and marsupial species, including *ATRX, SRY *and *SOX9*, although patterns of expression and regulation have drifted apart in the course of subsequent evolution [40].

## The mammalian paradigm

Sex determination in mammals has been more extensively studied than in any other species, most probably due to its direct relevance to human physiology and pathophysiology [10]. Accordingly, exploration of mammalian gonadal morphogenesis attracted for centuries a large number of investigators since the primordium retains a binary possibility to differentiate in both the male and female developmental pathway [41,42]. This can be achieved via the dosage- and time-dependent action of a series of sex-related genes [39-42]. Among these, the Y-linked gene *SRY *has been shown to be the primary male determinant that, in conjunction with other positive regulators, induces upregulation of several downstream genes such as *SOX9 *promoting the development of a functional testis [41]. In the XX gonadal primordium "negative regulators" operate during the appropriate window of time to repress "testicular" genes and promote "ovarian" analogues to secure the development of the female gonad [10,39-42].

A large number of genes have already been described in mammalian sex determination using the mouse as a model organism and many more are expected to be added in the process [10,39,40], since the related research constantly reveals new players in the complex network of reactions related to sex determination [41,42]. Even in the stage of the urogenital ridge, which forms the primordium of the gonad, adrenal, kidney and reproductive tract, the expression of several genes is considered crucial for subsequent development and normal sexual dimorphism [42-44]. These include, among others, *Fgf9 (Fibroblast growth factor 9), WT1 *(Wilm's tumor 1), *FtzF1/SF1 *(Fushi tarazu factor 1/steroidogenic factor 1 - sex steroid regulation), *Lim1(Lhx9), Emx2, M33, Pod1 *and, possibly, *Dmrt1 *[10,13,39-43]. Absence of any of their products at this stage, especially WT1, is inconsistent with further gonadal development and may also cause other malformations, e.g. affecting the adrenal gland and renal buds [41-44]. Genes of the *Wnt *family, such as *Wnt4*, may also participate in the regulation of epithelial organization and epithelial-mesenchymal interactions in the area of the gonadal primordium [42]. Afterwards, members of the insulin receptor family, as well as *GATA4/Fog2*, in conjunction with *WT1 *and *SF1*, are involved in *SRY *regulation by promoting its expression. [41,43,44].

In humans, formation of the testis is initiated in XY as well as XXY embryos, following the expression of *SRY *gene in the supporting somatic cell element of the gonadal primordium [45]. *SRY *acts during a narrow time window, ensures the commitment of these precursor cells to develop into Sertoli cells and therefore initiates testicular morphogenesis [45]. In fact, during testicular organogenesis, Sertoli cells are the first to differentiate in the gonadal primordium [45-47]. They originate from undifferentiated cells, *in situ*, and are transformed into large clear epithelial cells, which adhere to one another and encompass germ cells to form the seminiferous cords [46,47]. The SRY protein is localized within their nucleus and the window of its expression varies according to downstream gene expression reviewed in [42].

Both classes of therian mammals, placentals and marsupials, possess a Y chromosome and follow the *SRY *male sex-determination mechanism, thus implying that it must have emerged before their divergence 148 million years ago (MYA) [48].

Returning to evidence obtained via experimentation in rodents, several genes, including members of the *Sox E *family *Sox 8 *and *Sox9*, share a common HMG box, similar to that observed in *SRY*, which is considered necessary for their action at the molecular level [43,49]. The initial result of *Sry *expression is the upregulation of *Sox9 *gene expression in the differentiating male gonadal primordium [50]. Impairment of *Sox9 *interaction with DNA results to male - to - female sex reversal reviewed in [51] and ectopic expression of the ovarian markers Rspo1 and Foxl2 [52]. On the other hand, impairment of the nucleo-cytoplasmic translocation of SOX9 in XX gonads results in partial masculinization of the female gonad [53]. In fact, before testicular differentiation SOX9 protein is faintly expressed (50). In the male, during Sertoli cell differentiation SOX9 protein shuttles to the nucleus while is downregulated at 11.5 dpc in the XX gonad [53]. Antagonistic signals such as WNT4 (an inhibitor of FGF9) preserve the cytoplasmic localization status of SOX9 and suppress its expression, which is but one of their various actions promoting ovary development [54,55].

Besides mammals, members of the *Sox *group have been detected in various species of vertebrates, such as fish (*Sox9*) and monotremes (e.g. *Sox2 *and *Sox 14*) and this further emphasizes their importance as conserved components in genetic sex determination [56]. The observation of this gene family's evolutionary conservation adds one more credit to the multistage model of sex chromosome evolution, since *Sox3 *has been proposed as the autosomal ancestor of *SRY*, which places it among the chronologically first sex-related genes in the common evolutionary history of all vertebrates [57]. Interestingly, however, its expression in the emerging gonad has been confirmed in mice, humans, chicken and *Xenopus *but not in marsupials, a finding not consistent with the presumption of a uniform conserved role in mammalian sex determination [58].

In the female embryo, the Y chromosome is not present and, therefore, *SRY *is not expressed [45]. SOX9 maintain its cytoplasmic localization in the XX gonadal primordium and is down regulated by the formation of follicular cells in the ovary [54]. The genetic cascade regulating female reproductive system differentiation is not as extensively studied as in men, with only some players recognized so far, such as *Wnt4 *and *Rspo1 *(Rspondin1) [54,55]. Sex steroid production regulation is also important for the establishment of a normal female phenotype and it is mediated via SF1 expression and aromatase enzyme complex induction [16].

Two additional genes with a potential role in sex determination and differentiation are *DMRT1 *and *Stra8 *(stimulated by retinoic acid gene 8) [24-26,32,59,60]. The first has been already discussed in previous units as a conserved sex-related gene, bearing a DM domain originally studied in nematodes [24-26,32,59]. In humans, despite the report of XY sex reversal in cases of 9p chromosome deletions originally attributed to the impaired action of *DMRT1 *or its homologue, *DMRT2*, a definite involvement in the sex determination circuit has not been proven [60]. S*tra8*, on the other hand, is expressed in female germ cells and facilitates their entry into meiosis, in an anterior to posterior direction [61,62]. However, it is also expressed in male germ cells postnatally, thus making its expression sexually dimorphic, rather than sex-specific [61,62].

## Steroids in sex determination: Yet another common element?

Hormonal regulation of sex determination is a vast research field in modern reproductive endocrinology [62]. In fact, recent advances have resulted in a more generalized study of sexual dimorphism, with the discovery that differences expand to far more than the reproductive organs, including visceral tissues and the brain [62,63]. The study of sex steroid concentrations and the presence of their receptors in various parts of the CNS have already been attempted in various species, including mammals and reptiles [62-64]. After all, the role of androgens and estrogens in sexual differentiation in vertebrates is a classic concept that modern research data has only supported and expanded, rather than criticized [16]. For instance, aromatase regulation appears to be the final target in the sex determination circuit of several turtles [65]. This has been proven by the experimental work of C. Pieau and colleagues, using aromatase inhibitors to effectively block feminization of the embryos [65]. Relevant data is also available for sex reversal (at the post primary sex determination level) in fish and amphibians, while hormonal manipulations in avians and mammals usually result in intermediate and abnormal sex phenotypes [16].

Other scientists have even attempted to suggest sex steroids as a driving force in X-Y evolution [66]. According to such a hypothesis, androgens may be a major regulator of X-Y differentiation, acting as an evolutionary stress factor due to the induction of reproductive failure [66]. In particular, it is known that increased testosterone may be beneficial for fertility, but constant exposure to high quantities may result in spermatogenic arrest [66,67]. In response to such a steroid-induced pressure, the *DAZ *gene of the Y is proposed to have appeared 30-40 MYA as a means to maintain spermatogenesis and has remained on modern Y chromosomes as a conserved domain ever since [67].

In females, increased testosterone levels may have also caused evolutionary pressure (if associated with reproductive failure) and limited the total population, as only few of them survived and transferred their DNA in next generations, a process detected by mitochondrial DNA comparative studies [66-68]. This is an example of the bottleneck phenomenon, and due to its reference to females, it has been described as the mitochondrial Eve hypothesis [66-68]. A number of studies in comparative genomics seem to support this theory [69]. Increased testosterone levels acting in descendants of these women may have resulted in a second wave of evolutionary pressure, counteracted by a duplication of the DAZ gene, about 50.000-200.000 years ago, one again promoting the maintenance of spermatogenesis in male offspring [70]. These repetitive stages of evolutionary pressure and limitation of the total population may explain the large-scale homology of the regions of the Y chromosome among all modern males (Adam phenomenon) [71]. Failure to provide sufficient evidence, such as the description of all the genes affected by androgens, their exact importance for male fertility and the degree of their conservation among modern men has not allowed to adequately verify the validity of this theory to this date. Moreover, sex differentiation may also involve other, non-hormonal elements, including immunological parameters and paracrine messages/cytokines and therefore, sex steroids may not exhibit their full potential in simplified *in vitro *studies [72,73]. Nevertheless, their presence is well established in the sex differentiation of all vertebrate classes, proving their conserved evolutionary role in sex evolution (Table 1).

## Cellular-level sex determination and epigenetics: Invertebrate-only strategies or common mechanisms?

Invertebrates have been shown to possess quite distinct mechanism of sex determination compared to those of vertebrates. In particular, the genes examined so far do not bear homology or functional association to those of vertebrates and steroids, although present, have not been clearly associated with sex-specific actions [14,74-77]. Sex chromosomes may be present, but no structural association to their analogues in vertebrates can be traced [14,75-77].

Another significant observation in invertebrate sex determination refers to its establishment (in worms) at a cellular level, rather than the whole organism [77]. As previously discussed, recent evidence from birds indicates that a similar strategy of sex determination at a tissue level may be present in these species as well [38]. In an attempt to justify these observations, some researchers have proposed that sexual dimorphism in general is associated with selective cell proliferation [74]. This hypothesis is based on the comparative observation of male and female gonadal development in different successive stages and for a number of different model organisms [74]. If this is indeed a common strategy, it could be the result of sex steroid regulation (via induction of sex - specific growth factors) [16]. The description of several conserved sex-related genes in various species may also support this view [10,13]. Alternatively, sexually dimorphic development could also be the result of a steroid-independent pathway of mitotic induction, implicating other growth and differentiation factors, such as those detected in invertebrates [75-77].

Invertebrates have been also shown to implicate chromatin level interactions in their sex determination [75-77]. Their sex determination pattern typically involves X chromosome activation and a "counting process" that estimates the X: autosome chromosome ratio within the whole organism (fruitflies) [75,76] or any given cell autonomously (e.g. worms) [77]. Epigenetic regulation of the sexual phenotype has also clearly been proven [75-77]. In particular, the sex-specific alternative splicing products of key genes, namely *dsx, fru *and *tra*, may influence further DNA replication and/or transcription [75-77]. The detection of the evolutionary history of this mechanism may allow its categorization as an invertebrate exclusivity [14,78,79].

## Conclusion

Sex determination remains an intriguing field of study, with various open questions that pose a challenge for researchers in the field. Sequencing and comparative genetics data seem to suggest that a common evolutionary origin for all mammalian sex determination strategies is most probable and this is depicted in common main regulators shared among modern organisms (e.g. *SRY, DMRT*) [38-42]. This observation may also imply the existence of similarities in these species' reproductive physiology, at least during the crucial developmental phenomena of organogenesis *in utero*[46,47]. Since this is considered a period of great significance for reproductive health in the adult life, the exploration of the regulatory networks involved (with the aid of model organisms and advanced techniques), may allow the better understanding of infertility issues and the development of novel, more efficient therapeutic interventions in future.

In terms of sex determination pathways' evolution, evidence seems to accumulate in favor of a more unified approach that incorporates all currently observed strategies (ESD, including TSD and GSD, including sex chromosome formation) [2,7,8,10]. In particular, it seems possible that environment is the driving force behind sex development and evolution, as it is also the driving force for any other level of evolution (via sex and natural selection) [2]. What makes the difference is the extent to which this interaction is directly mediated via non-genomic elements (as is probably the case in ESD-TSD) or has been chronically incorporated in more complex genetic networks involved in various steps of the sex determination--differentiation process (GSD, sex chromosomes) in the course of evolutionary history [2]. It is also probably true that sex evolution has also been affected by phenomena such as sex selection and drift and such incidents may have also left an "imprint" in modern sex determination patterns [10]. In this perspective, the proper question should therefore probably no longer be whether sex is controlled by environment or genes, but rather, in what way and to what level these factors contribute to its regulation in any given context.

Interestingly, as more experimental data is gathered and the complicated molecular pathways involved in sex determination become more evident, the need to re-examine sex itself emerges. Several decades ago, scientific evidence achieved the description of hierarchical levels of sex establishment, ranging from chromosome content to gonadal development, secondary trait differentiation and, finally, mental--cognitive and behavioral adaptations, thus adopting the concept of sexual dimorphism in a truly holistic manner [80]. Now that the first steps in this process are becoming better understood and similarities and differences among the various species clearly elucidated, it might be the time to take a step forward and view sex in a more global context: similar tools (e.g. conserved genes, locally and temporally selective sex steroid production and action) have been used and controlled in a different way (ESD, TSD, GSD, sex chromosomes and combinations of the above) to surpass specific adaptation challenges (environmental pressure) in the course of evolution [80].

## Abbreviations

**DAZ: **Deleted in AZoospermia; **DMRT1: **Doublesex- and mab-3-related transcription factor 1; **DMY: **DM domain of the Y chromosome; **ESD: **Environmental sex determination; **FOXL2: **Forkhead box L2; **FTzF1: **Fushi tarazu factor 1; **GSD: **Genetic sex determination; **her: **Hermaphrodite; **HER-1: **Hermaphrodite 1; **HMG: **High-mobility group; **MYA: **Million years ago; **ovol1: **Ovo-like 1; **Rspo1: **Rspondin1; **SF1: **Steroidogenic Factor 1; **SOX9: **SRY-related high-mobility group box 9; **SRY: **Sex determining region of the Y chromosome; **Stra8: **Stimulated by retinoic acid gene 8; **sxl: **Sex-lethal; **TRA-1: **Transformer 1; **TSD: **Temperature-dependent sex determination; **TSP: **Thermosensitive period; **WNT4: **Wingless Int 4; **WT1: **Wilm's tumor 1

## Competing interests

The authors declare that they have no competing interests.

## Authors' contributions

The three authors have contributed equally in all the steps of the preparation of the submitted manuscript. In particular, the bibliographical research, the compilation of the first draft and the proofreading and editing processes were undertaken by the contributing authors in a series of relevant meetings in the Department of Histology and Embryology, Medical School, Athens University, Greece. All authors read and approved the final manuscript.
